# Scattering exceptional point in the visible

**DOI:** 10.1038/s41377-023-01282-4

**Published:** 2023-09-15

**Authors:** Tao He, Zhanyi Zhang, Jingyuan Zhu, Yuzhi Shi, Zhipeng Li, Heng Wei, Zeyong Wei, Yong Li, Zhanshan Wang, Cheng-Wei Qiu, Xinbin Cheng

**Affiliations:** 1 MOE Key Laboratory of Advanced Micro-Structured Materials, Shanghai, 200092 China; 2 Institute of Precision Optical Engineering, School of Physics Science and Engineering, Tongji University, Shanghai, 200092 China; 3 Shanghai Institute of Intelligent Science and Technology, Tongji University, Shanghai, 200092 China; 4 Shanghai Frontiers Science Center of Digital Optics, Shanghai, 200092 China; 5 Shanghai Professional Technical Service Platform for Full-Spectrum and High-Performance Optical Thin Film Devices and Applications, Shanghai, 200092 China; 6 Department of Electronic Science and Technology, Tongji University, Shanghai, 201804 China; 7 Department of Electrical and Computer Engineering, National University of Singapore, Singapore, 117583 Singapore; 8 Institute of Acoustics, School of Physics Science and Engineering, Tongji University, Shanghai, 20092 China

**Keywords:** Sub-wavelength optics, Metamaterials

## Abstract

Exceptional point (EP) is a special degeneracy of non-Hermitian systems. One-dimensional transmission systems operating at EPs are widely studied and applied to chiral conversion and sensing. Lately, two-dimensional systems at EPs have been exploited for their exotic scattering features, yet so far been limited to only the non-visible waveband. Here, we report a universal paradigm for achieving a high-efficiency EP in the visible by leveraging interlayer loss to accurately control the interplay between the lossy structure and scattering lightwaves. A bilayer framework is demonstrated to reflect back the incident light from the left side ( | *r*_−1_ | >0.999) and absorb the incident light from the right side ( | *r*_+1_ | < 10^–4^). As a proof of concept, a bilayer metasurface is demonstrated to reflect and absorb the incident light with experimental efficiencies of 88% and 85%, respectively, at 532 nm. Our results open the way for a new class of nanoscale devices and power up new opportunities for EP physics.

## Abstract (web summary)

We report a universal paradigm for achieving a high-efficiency EP in the visible by leveraging interlayer loss to accurately control the interplay between the lossy structure and lightwaves.

## Graphical abstract

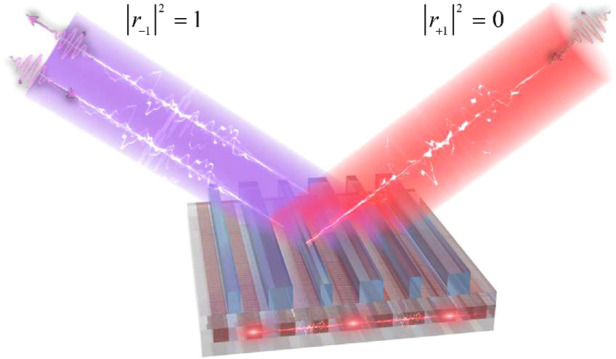

## Introduction

Exceptional point (EP)—characteristic of non-Hermitian systems—refers to the spectral singularity of open systems whose eigenvalues and their corresponding eigenvectors degenerate simultaneously^1–3^. Non-Hermitian systems have numerous alluring optical properties at EPs^4–7^ and have attracted extensive attention because of their great prospects in applications such as optical sensing^8–10^, integrated optics^11^, and other fields^12–17^. In the early stage of implementing EPs, the optical gain and loss were integrated as non-conservative components in a simple one-dimensional transmission system to achieve Eps^18,19^, while the optical gain often leads to complex and unstable systems. EPs can also be observed by designing complex permittivity in all-passive systems, which inspires the study of EP-related phenomena^20^, for instance, the one-way cloaking^21–23^. However, one-dimensional transmission systems have limited capabilities in controlling lightwaves^24–28^.

**Fig. 1:**
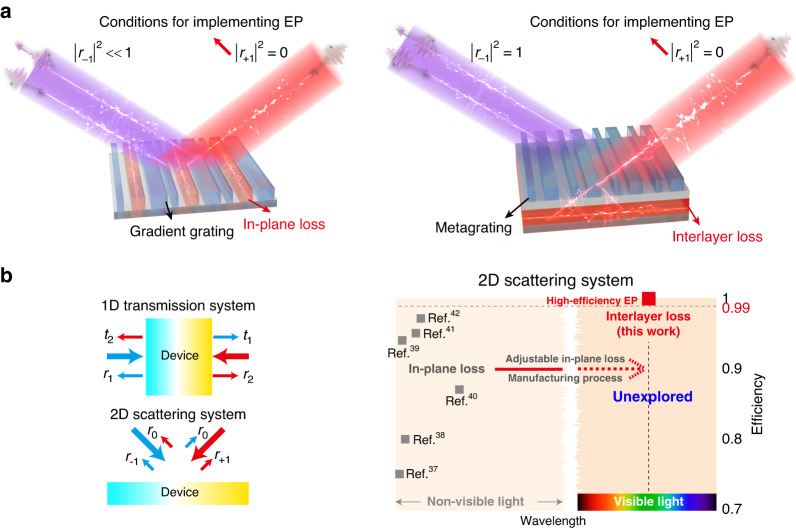
Models of high-efficiency scattering exceptional point. **a** The physical model and principle of the optical non-Hermitian metasurfaces operating at an EP based on the in-plane loss and interlayer loss. **b** The concept of 1D transmission system and 2D scattering system. Comparative overview of the non-Hermitian metasurface based on in-plane loss and interlayer loss

Metasurface, a class of artificial materials that transcends natural materials through the orderly design of subwavelength structures^29–36^, has become a new platform to realize complex optical Eps^27,37–46^. The reflection/transmission or diffraction of the metasurface can be described by scattering matrix^47–50^. For a two-port scattering metasurface system (Fig. 1a), the scattering matrix describing the diffraction properties of the system is $$\left[\begin{array}{cc}{r}_{0} & {r}_{-1}\\ {r}_{+1} & {r}_{0}\end{array}\right]$$ (*r*_0_ is the specular reflection, *r*_−1_ and *r*_+1_ are retroreflections). When $\sqrt{{r}_{-1}{r}_{+1}}=0$, the eigenvalues of the scattering matrix are degenerate and an EP occurs. A common way to implement scattering EP is regulating the loss of the gradient metasurfaces by introducing a specific loss in a unit cell^37–39^. Recent works have demonstrated non-Hermitian metasurfaces composed of a loss-assisted supercell (Fig. 1a) in the microwave range operating at an EP^40–42^. However, direct extensions of in-plane loss in a gradient metasurface from non-visible waveband to visible light remain a formidable challenge, since the adjustable in-plane loss and corresponding manufacturing process in the visible are lacking^44^. Further, the complex and rebellious interplay between in-plane lossy structure and lightwave restricts optical efficiency. Therefore, achieving high-efficiency EP at optical non-Hermitian metasurface is still a challenging task in photonics. Notably, two-dimensional scattering systems operating at EPs in the visible are unexplored.

Here, we propose a strategy to use the interlayer loss to accurately control the interplay between the lossy structure and lightwaves via regulating the multiple scattering process using a bilayer architecture (Fig. 1a). A high-efficiency scattering non-Hermitian metasurface operating at an EP in the visible was demonstrated for the first time (Fig. 1b) to reflect back the incident light with left-side incidence ( | *r*_−1_ | >0.999) and absorb the incident light with right-side incidence ( | *r*_+1_ | <10^−4^). As a proof of concept, we design a bilayer metasurface composed of TiO_2_ metagrating and Si subwavelength grating: the metagrating in the upper layer achieves the directional regulation of lightwave, and the lossy subwavelength grating in the lower layer achieves an adjustable absorption. The fabricated sample is experimentally demonstrated to reflect and absorb incident light with efficiencies of 88% and 85%, respectively, at 532 nm. Our work exhibits a universal paradigm for achieving a high-efficiency EP in a two-port scattering system, which would inspire more novel photonic devices for wave manipulation.

## Results

### Models and theory

Traditional non-Hermitian metasurface utilizes *N* subunits in one period to realize a gradient phase, as shown in Fig. 1a. When the lightwave impinges the metasurface with opposite directions, the metasurface would impose a pair of identical phase gradients (*dφ*/*dx*) to the lightwave, leading to the asymmetric reflection or transmission, also known as the generalized Snell’s law^51^. In the case of extremely asymmetric responses, if the lossy structure is introduced, the lossy structure will absorb the lightwave selectively^40^. Particularly, the perfect absorption will be achieved by elaborately designing the extinction coefficient and geometric parameters of the lossy structure, such that the two-port scattering metasurface system reaches an EP^39,42^. However, the combination of lossless and lossy structures makes it difficult to regulate the interplay between lossy structure and lightwaves completely when lightwave impinges the metasurface from opposite directions. Therefore, traditional in-plane lossy non-Hermitian metasurfaces cannot exhibit high-efficiency EPs. It is of the utmost importance in developing new design methodology to accurately control the interplay between lossy structure and lightwave for creating high-efficiency optical EPs.

The high-efficiency meta-system at an EP is essentially a wave vector-dependent perfect retroreflector and absorber, as shown in the right of Fig. 1a. The key issue for achieving a high-efficiency is controlling the interplay between lossy structure and lightwaves precisely: when the lightwave impacts from one side, the perfect retroreflector requires no interplay between the lossy structure and lightwave; when the lightwave impacts from the opposite side, the perfect absorber requires full interplay between the lossy structure and lightwave. In-plane lossless and lossy hybrid structures can never completely isolate the scattering of lightwaves from opposite directions. Hence, we propose the strategy to use an asymmetric metagrating and an interlayer lossy structure to accurately control the interplay between the lossy structure and the lightwave. On the one hand, the metagrating in the upper layer achieves a perfect retroreflection for the incident lightwave from one side by regulating the interference of Bloch-mode supported by the metagrating. On the other hand, the lossy structure in the lower layer achieves total absorption for the incident lightwave from the other side by adjusting the absorption coefficient of the lossy structure via a complex multiple scattering process.

### Design of high-efficiency scattering EP

**Fig. 2:**
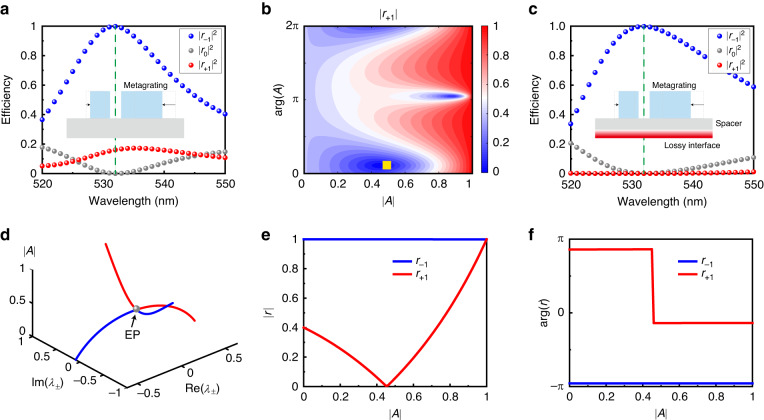
Design methodology for high-efficiency scattering EP based on interlayer loss. **a** The spectra and schematics of the asymmetric metagrating. **b** The amplitude of non-specular reflection coefficients *r*_+1_ by varying amplitude and phase of complex coefficient *A*. **c** The spectra and schematics of the high-efficiency optical non-Hermitian meta-system at EP. The |*r*_−1_| is larger than 0.999 and |*r*_+1_| is less than 10^-4^. **d** Trajectories of eigenvalues *λ*_±_ with the evolution of |*A* | . **e** The amplitude of non-specular reflection coefficients *r*_−1_ and *r*_+1_ by varying |*A* | . **f** The phase of non-specular reflection coefficients *r*_−1_ and *r*_+1_ by varying |*A* |

The high-efficiency meta-system at an EP is composed of an asymmetric metagrating for perfect retroreflection and a lossy structure for perfect absorption. The perfect retroreflection is based on Littrow grating by controlling the interference of Bloch-modes^52,53^ with two-groove grating configuration. Firstly, a high-efficiency Littrow grating is designed by leveraging the efficiency spectrum verse geometric parameters as shown in Section 1 of Supporting Information. When the geometric parameters of the two-groove grating are selected, the retroreflection amplitudes are approaching unity as shown in Section 1 of Supporting Information. The Littrow grating has exactly the same response when lightwaves impinge grating from two symmetrical directions given as $$\left[\begin{array}{cc}{r}_{0} & {r}_{-1}\\ {r}_{+1} & {r}_{0}\end{array}\right]=\left[\begin{array}{cc}0 & 1\\ 1 & 0\end{array}\right]$$. To break the symmetrical response of the grating, we moved the positions of the two grooves a certain distance, as shown in the inset of Fig. 2a. We can thus break the symmetric response while maintaining the retroreflection amplitude *r*_−1_ of 1 by changing the grating parameters locally. As exhibited in the spectra of Fig. 2a, the metagrating completely reflects the incident light from the left and partially reflects the incident light from the opposite direction. In this case, the scattering matrix of the metagrating develops to $$\left[\begin{array}{cc}{r}_{0} & {r}_{-1}\\ {r}_{+1} & {r}_{0}\end{array}\right]=\left[\begin{array}{cc}0 & 1\\ x & 0\end{array}\right]$$, where *x* ≫ 0 at the designed wavelength of 532 nm.

To further realize high efficiency (*x* = 0), a composite structure with a lossy interface under the spacer (the inset of Fig. 2c) is deployed to support the lightwave to undergo a multiple scattering process. The final response of the system can be described as

1 $$\left[\begin{array}{cc}{r}_{0} & {r}_{-1}\\ {r}_{+1} & {r}_{0}\end{array}\right]=R+{T}^{{\prime} }{\left(I-\left[\begin{array}{cc}A & 0\\ 0 & A\end{array}\right]{R}^{{\prime} }\right)}^{-1}\left[\begin{array}{cc}A & 0\\ 0 & A\end{array}\right]T$$

Here, *R* and *R’* are the reflection coefficients of the metagrating as a plane wave is being incident from air and spacer, while the *T* and *T’* are the transmission coefficients as a plane wave is being incident from air and spacer. *A* is a complex coefficient describing the effect of absorption/reflection amplitude and phase accumulation during the propagation, which is completely determined by the underlying lossy structure and spacer (detailed equations are given in Section 2 of Supporting Information). Although the metagrating itself cannot directly construct the scattering matrix required by high-efficiency optical non-Hermitian meta-system at EP, the multiple scattering process would enhance the ability of the metagrating in controlling lightwaves, thus expanding the range of the scattering matrix. Because the metagrating will reflect all the incident light back when it comes from the left (as shown in Fig. S3 of Supporting Information), we only need to consider the effect of the lossy structure on *r*_+1_. As a result, the behaviors of lightwaves from opposite directions are completely isolated. Figure 2b shows the amplitude of *r*_+1_ versus the amplitude and phase of complex coefficient *A*. With a specific combination of amplitude and phase, the amplitude of *r*_+1_ can be controlled dynamically from near 0 to 1. When proper parameters such as (0.4538, 0.3412) for the amplitude and phase as marked by a yellow square are selected, the |*r*_+1_| = *x* is less than 10^−4^ (near zero), which means 100% absorption. In this case, the scattering matrix of the meta-system develops to $$\left[\begin{array}{cc}{r}_{0} & {r}_{-1}\\ {r}_{+1} & {r}_{0}\end{array}\right]=\left[\begin{array}{cc}0 & 1\\ 0 & 0\end{array}\right]$$. Figure 2c demonstrates a wave vector-dependent perfect retroreflector and absorber.

The eigenvalues of the scattering matrix of a non-Hermitian system are ${\lambda }_{\pm }={r}_{0}\pm \sqrt{{r}_{+1}{r}_{-1}}$. Figure 2d shows the evolution trajectories of eigenvalues with the amplitude variation of *A* while the phase of *A* equals 0.3412. Distinctly, when the amplitude of *A* is at 0.4538, an EP appears as the eigenvalues coalesce into a real number value, which is similar to what was observed in acoustic and optical systems^39,42^. With the amplitude of *A* increasing, the amplitude of *r*_+1_ initially decreases and then increases, while the amplitude of *r*_−1_ is stably greater than 0.998, as shown in Fig. 2e. The amplitude of *r*_+1_ equals 0 when the amplitude of *A* is at 0.4538, indicating that the extraordinary retroreflection from the right port is completely suppressed. At the same time, the phase of *r*_+1_ experiences an abrupt change of *π* at the EP and the phase of *r*_−1_ is virtually constant when the amplitude of *A* changes, as shown in Fig. 2f. From the above analysis, it is not difficult to find that we have theoretically achieved a high-efficiency optical non-Hermitian system at an EP.

### Experiments

**Fig. 3:**
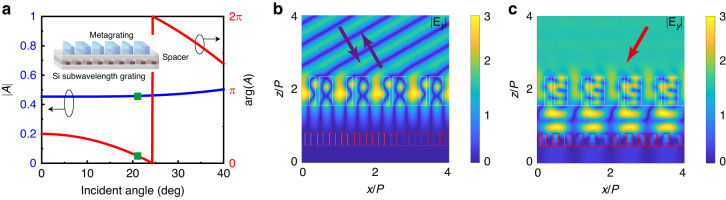
The bilayer high-efficiency optical non-Hermitian metasurface at EP composed of metagrating and subwavelength Si grating. **a** The spectra of the Si subwavelength grating and schematic diagram of the bilayer metasurface. The spectra are calculated with the incident medium being the spacer. **b** The electric field distributions of the double-layer metasurface with left-side incidence. **c** The electric field distributions of the double-layer metasurface with right-side incidence

As a proof of concept, we propose to use a Si subwavelength grating to realize the desired absorption/reflection, and an elaborately designed spacer to realize the phase accumulation, as shown in Fig. 3a. Although the resonant grating can also implement the desired absorption/reflection, the Si subwavelength grating not only simplifies the design, but also relaxes the alignment requirements in the fabrication. The phase accumulation during the propagation can be realized directly by choosing a spacer with a proper thickness (with a thickness larger than a quarter-wavelength of light inside the spacer). Figure 3a shows the amplitude and phase of *A* verse the incident angle when the duty cycle of Si grating is 0.468 and the thickness of the spacer is 397.5 nm. It is worth noting that the transmittance is very small and can be neglected. The amplitude and phase of *A* are 0.458 and 0.354, respectively, at an incident angle of 20.6° as marked by a green square, corresponding to the requirement of the high-efficiency optical non-Hermitian metasurface at an EP. In the design process, what we care about is the amplitude and phase of the structure in the lower layer, that is coefficient *A*. Therefore, there is no limitation for the period or other matching problems. As long as it satisfies the requirements of Fig. 2b, it is a good candidate for high-efficiency non-Hermitian metasurface. Then, a high-efficiency optical non-Hermitian metasurface operating at EP, which is composed of the TiO_2_ metagrating and Si grating, is obtained. Detailed parameters of the bilayer metasurface are listed in Fig. S4 of Supporting Information.

The electric field distributions of the non-Hermitian metasurface are exhibited in Fig. 3b, c to visually manifest the realization of high-efficiency EP: when impinging the metasurface from the left direction, the lightwave is bounded within the metagrating, leading to no interplay between Si grating and lightwave; when impinging the metasurface from the right-hand side, the lightwave penetrates the metagrating and is absorbed by the Si grating. In short, the high-efficiency optical non-Hermitian metasurface at EP is achieved via regulating the interplay between lossy structure and lightwave precisely by means of elaborately designing the TiO_2_ metagrating and Si grating.

**Fig. 4:**
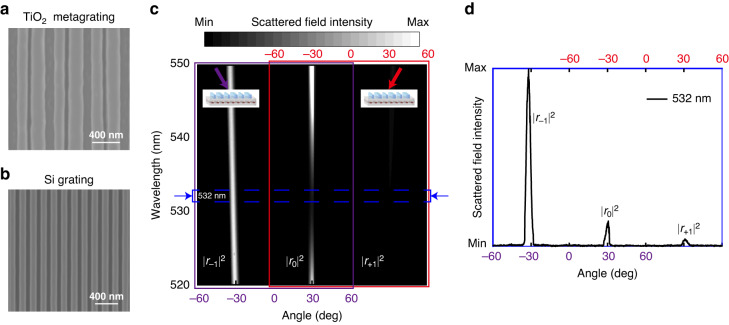
Experimental results of the high-efficiency optical non-Hermitian metasurface at EP. Top view (**a**) and back view (**b**) of the sample. The upper layer is TiO_2_ metagrating and the lower layer is Si grating. **c** The far-field scattered light from all angles of the sample with incident angles of 30° and −30°. **d** The experimental measured far-field scattered light from all angles with incident angles of 30° and −30° at 532 nm

A preparation process for bilayer microstructures was used to fabricate the sample as shown in Methods. Figures 4a, b show the top view and back view of the sample. The upper layer is TiO_2_ metagrating while the lower layer is Si grating. The angle-resolved spectrum system in the micro-region (as shown in Fig. S5 of Supporting Information) from Ideaoptics Inc. was employed in a darkroom to verify the spectral property of the non-Hermitian metasurface. In the test, the angle resolution was realized by focusing the reflective beam in the Fourier plane of the lens. Then, the far-field scattered lightwaves from all angles of the sample were detected. We conducted two separate tests with incident angles of 30° and −30° as shown in the purple and red boxes of Fig. 4c. When the incident light impacted from the left, most of the light was reflected by the sample to the incident path. When the incident light came from the right, there was very little retroreflection light. The far-field scattered light from all angles at 532 nm was shown in Fig. 4d. The scattered field intensity of *r*_−1_ is 7.2 times stronger than *r*_0_, while *r*_0_ is 3.7 times stronger than *r*_+1_. In general, the scattered field intensity ratio between *r*_−1_ and *r*_+1_ is about 26.7:1. In this case, the fabricated sample demonstrated reflection efficiencies of 88%, 12%, and 3% for *r*_−1_, *r*_0_, and *r*_+1_, respectively. The retroreflection and absorption efficiencies were 88% and 85%, respectively, for the left- and right-side incidence at 532 nm. The degeneration of experimental results compared with designed results comes from the fabrication tolerance because the optical response of non-Hermitian metasurface operating at EP is very sensitive to structural parameters. The efficiency could be further increased to the designed efficiency by optimizing the preparation process.

## Discussion

The most important issue to realize the high-efficiency optical non-Hermitian metasurface at EP is regulating the interplay between the lossy structure and lightwaves. By utilizing the control ability of metagrating in the electromagnetic wave, an asymmetric response can be obtained. Nonetheless, it does not meet the demand of a non-Hermitian metasurface operating at an EP. We propose an exquisite strategy to use an interlayer loss to accurately control the system loss. A multiple scattering process is stimulated by a bilayer configuration to enhance the ability in controlling lightwaves. We have theoretically achieved a high-efficiency optical non-Hermitian system at an EP by delicately regulating the interlayer loss. As a proof of concept, a subwavelength lossy grating is employed to realize the specific absorption and a bilayer high-efficiency optical non-Hermitian metasurface at EP is demonstrated. In fact, other structures like resonant structures, absorbing films and so forth, are also potential candidates for interlayer loss. Hence, the design of the high-efficiency optical non-Hermitian metasurface at EP using the proposed methodology is not cumbersome. However, due to the high sensitivity of non-Hermitian systems, their performance is extremely sensitive to structural parameters, putting forward extremely stringent requirements for the preparation and testing, which is the major difficulty for the high-efficiency optical non-Hermitian metasurface operating at EP. Besides, if using the nonlinear material to realize the specific absorption or increasing the power of the input laser, the nonlinear EP or other nonlinear effects may be able studied^54,55^.

In conclusion, to the best of our knowledge, we realized the first high-efficiency scattering non-Hermitian metasurface operating at EP in the visible by completely isolating the behavior of lightwaves from opposite directions. An interlayer loss is introduced to accurately control the system loss while a metagrating is used to realize a perfect retroreflector. The interlayer loss is precisely engineered by changing the duty cycle of a subwavelength lossy grating. We demonstrate a high-efficiency scattering non-Hermitian metasurface at 532 nm using our nontrivial methodologies in both design and experiment. Our work paves a new avenue toward the design of versatile optical metasurface platforms involving the EP or higher-order EP, which may inspire more functional photonic devices for wave manipulation.

## Materials and methods

### Sample fabrication

A preparation process for bilayer microstructures was used to fabricate the sample. Firstly, the Si was deposited on fused silica substrates using ion beam sputtering deposition in Vecco Spector System. The ion-beam voltage and ion-beam current were set as 900 V and 400 mA, respectively, with a base pressure of 4 × 10^−4^ Torr. The deposition rate of Si was 1.13 Å·s^–1^. Then, a layer of positive electron beam resist (EBR) (ZEP-520A) with a thickness of 120 nm was spin-coated on the coating, followed by baking for 5 min at 180 °C on a hot plate. A 10-nm chromium was then evaporation coated to avoid charging effects during the writing process. Next, target patterns were defined on the resist layer by EBL on a Raith 5200 system (100 kV, 3 nA). After EBL, the chromium was removed by chromium remover and the EBR was developed in amyl acetate for 1 min under gentle agitation. After that, we got the Si gratings on Oxford PlasmaPro100 Cobra with ICP-RIE (inductively coupled reactive ion etching) using a mixture of SF_6_ and CHF_3_ gas (15 and 50 sccm, respectively) at a pressure of 15 mTorr, substrate bias of -380 V, ICP power of 800 W. Etch rate was approximately 1 nm·s^–1^. A layer of negative optical photoresist HSQ (hydrogen silsequioxane) of thickness 500 nm was spin-coated and exposed by the EBL process to cover the Si gratings. The thickness was corrected to the target value using ICP-RIE. A layer of positive EBR (PMMA) with a thickness of 450 nm was spin-coated on the HSQ, and exposed by the EBL process to get the negative metagrating patterns. The PMMA was developed with MIBK:IPA = 3:1 for 1 min under gentle agitation. The TiO_2_ is deposited directly onto the exposed EBR through atom layer deposition (ALD) at a low temperature (90 °C) to avoid the glass transition of EBR. During the ALD process, a standard two-pulse system of water and TiCl_4_ precursor was used with a 0.05-s water pulse followed by a 3-s delay and a 0.4-s TiCl_4_ pulse followed by a 4-s delay, to ensure the complete reaction and full removal of the excessive precursors and reaction byproducts. The deposition rate was about 0.07 nm/cycle. This process ultimately left a nearly blanket film of TiO_2_ covering the entire EBR patterns which must be removed to expose individual patterns. This film was removed with ICP-RIE using a mixture of CHF_3_ and Ar gas (20 and 80 sccm, respectively) at a pressure of 15 mT, substrate bias of -380 V, ICP power of 800 W. Etch rate was approximately 1.67 nm·s^−1^. After the covering film was removed, we used oxygen plasma to remove the remaining photoresist and placed the samples in PG Remover for 24 h to make sure the EBR is removed completely.

### Supplementary information

Supplementary Information for Scattering exceptional point in the visible

## Data Availability

Data underlying the results presented in this paper are available from the corresponding author upon reasonable request.
