# Supplementary material for: Scattering exceptional point in the visible
Source: Light Sci Appl. 2023 Sep 15;12:229. doi: 10.1038/s41377-023-01282-4 (PMC10504253; doi:10.1038/s41377-023-01282-4)
Supplement: Supplementary file 1 — Supplementary Information for Scattering exceptional point in the visible [file 41377_2023_1282_MOESM1_ESM.docx]

Supplementary Information for

Scattering exceptional point in the visible

Tao He^1,2,3,4,5,6^†, Zhanyi Zhang^1,2,3,4,5^†, Jingyuan Zhu^1,2,3,4,5^†, Yuzhi Shi^1,2,3,4,5^, Zhipeng Li^7^, Heng Wei^7^, Zeyong Wei^1,2,3,4,5^, Yong Li^8^, Zhanshan Wang^1,2,3,4,5^, Cheng-Wei Qiu^7^*, Xinbin Cheng^1,2,3,4,5^*

^1^MOE Key Laboratory of Advanced Micro-Structured Materials, Shanghai 200092, China.

^2^Institute of Precision Optical Engineering, School of Physics Science and Engineering, Tongji University, Shanghai 200092, China.

^3^Shanghai Institute of Intelligent Science and Technology, Tongji University, Shanghai 200092, China.

^4^Shanghai Frontiers Science Center of Digital Optics, Shanghai 200092, China.

^5^Shanghai Professional Technical Service Platform for Full-Spectrum and High-Performance Optical Thin Film Devices and Applications, Shanghai 200092, China.

^6^Department of Electronic Science and Technology, Tongji University, Shanghai 201804, China.

^7^Department of Electrical and Computer Engineering, National University of Singapore, Singapore 117583, Singapore.

^8^Institute of Acoustics, School of Physics Science and Engineering, Tongji University, Shanghai 20092, China.

†These authors contributed equally to this work.

*Corresponding author:

Email: chengwei.qiu@nus.edu.sg (C.-W.Q.); [chengxb@tongji.edu.cn](mailto:chengxb@tongji.edu.cn) (X.C.)

1. **The design method for high-efficiency Littrow grating.**

It is widely known that a two-groove grating can realize high-efficiency retroreflection in the Littrow mounted by controlling the interference of Bloch-modes^1, 2^. The critical factors affecting the interference of Bloch modes are the coupling coefficients at the upper and lower interface, and the phase accumulation in the grating. To be more precise, the phase accumulation is obtained via multiplying the effective refractive index by the propagation distance. In parallel, the coupling coefficients and phase accumulation are determined by the ridge widths (*w*_1_, *w*_2_) and height (*H*) of grating. Considering that the two-groove grating supports four propagation Bloch modes, it is difficult to control each mode analytically to achieve both no transmission and high-efficiency retroreflector. At the same time, the two-groove grating only has three free parameters. Therefore, the high-efficiency Littrow grating can be obtained easily by leveraging the efficiency spectrum verse (*w*_1_, *w*_2_, *H*) as shown in Fig. S1. When the parameters (94.8, 208.5, 391.9) marked by arrow are selected, the amplitude of retroreflection is near 1. The Littrow grating has exactly the same response when lightwaves impinge grating from two symmetrical directions, as shown in Fig. S1.


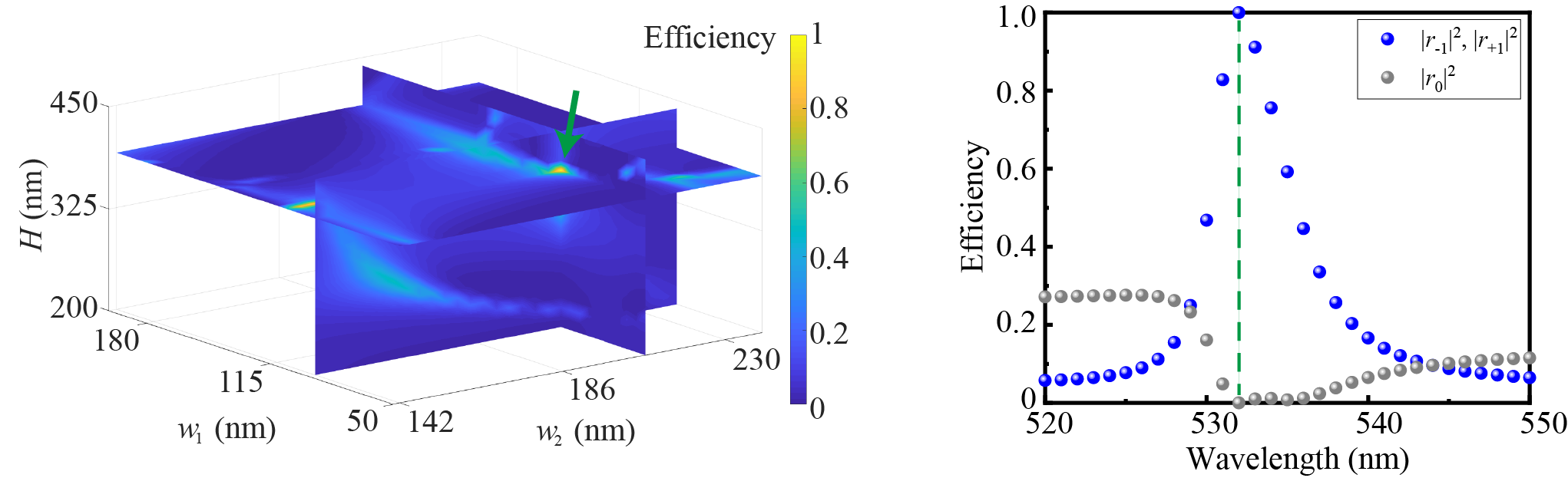


Fig. S1 The efficiency spectrum of high-efficiency Littrow grating.

1. **The multiple scattering process of a bilayer metasurface.**

In a general situation, as shown in Fig. S2, when incident light impinges a bilayer metasurface, some of the incident light will be reflected directly into air, which is recorded as *r*. The rest, that is transmitted light recorded as *t* will go through the metasurface into the spacer. The light in the spacer would undergo a phase accumulation during the propagation and be reflect by the interface of spacer and substrate. This propagation process can be written as $A=e^{ik_{2}h_{2}}R_{b}e^{ik_{2}h_{2}}$, where *R_b_* is the reflection coefficient of the interface. For a lossy interface without transmission, the reflection coefficient is also equivalent to absorption amplitude. Therefore, the *A* is a complex coefficient describing the effect of absorption or reflection amplitude and phase accumulation during the propagation, which is completely determined by the underlying lossy structure and spacer. As the light travels, partial light will go through the metasurface into the air, which is written as *χ=t*’*At.* The other light will reflect back and undergo the same process as the process described above. The light escapes from the spacer are *χρ=t*’*Ar*’*At*, *χρ*^2^*=t*’*Ar*’*Ar*’*At* … The *r* and *r*’ are the reflection coefficients of the metasurface as a plane wave is incident from air and spacer, while the *t* and *t*’ are the transmission coefficients as a plane wave is incident from air and spacer, respectively. From the above derivation, we can get the total reflection of the bilayer metasurface as follows: $r_{total}=r+\chi+{\chi\rho}^{1}+{\chi\rho}^{2}+\ldots=r+t^{'}\left( 1-Ar^{'} \right)^{-1}At$. Considering a multiport scattering system, all the reflection and transmission coefficients need to be transformed into a matrix form. Then, the expression of *r_total_* can be rewritten as $\left[ \begin{matrix} r_{0} & r_{-1} \\ r_{+1} & r_{0} \end{matrix} \right]=R+T^{'}\left( I-\left[ \begin{matrix} A & 0 \\ 0 & A \end{matrix} \right]R^{'} \right)^{-1}\left[ \begin{matrix} A & 0 \\ 0 & A \end{matrix} \right]T$, where *R*, *R*’, *T*, *T*’ are the matrix form of *r*, *r*’, *t*, *t*’.


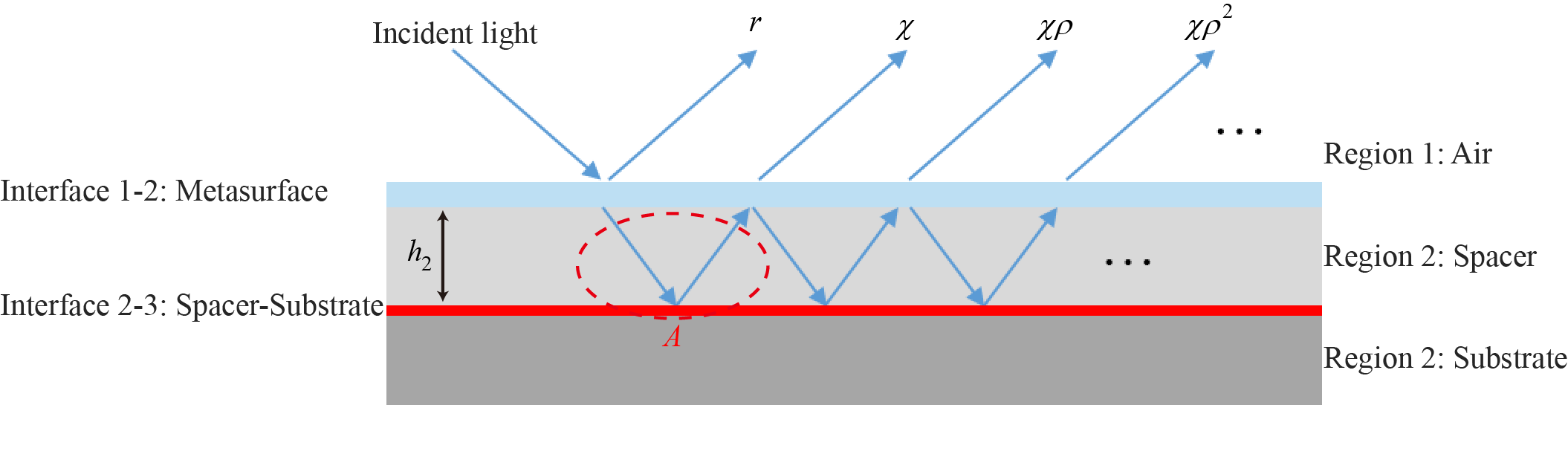


Fig. S2 The schematics of a bilayer metasurface with a lossy interface under the spacer, which exhibits multiple scattering process from geometric optics.


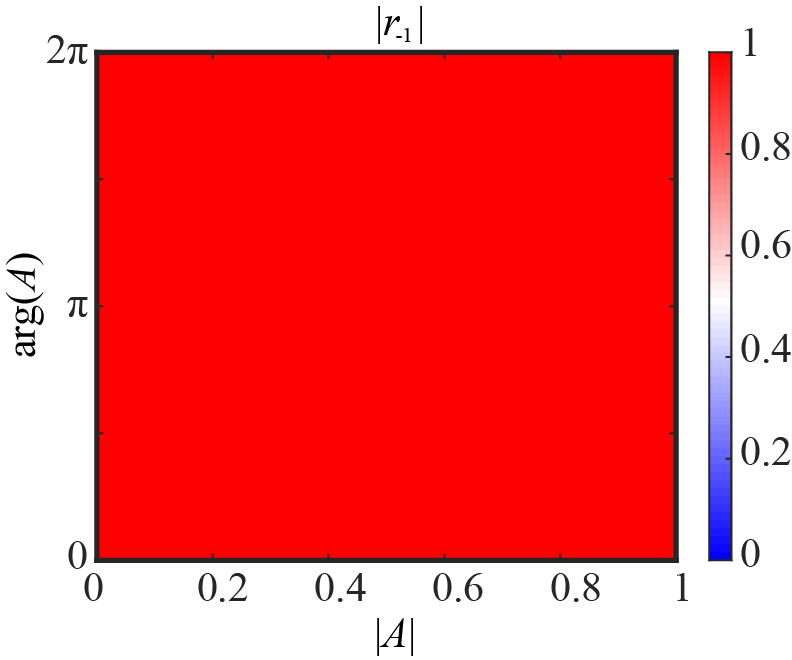


Fig. S3 The amplitude of non-specular reflection coefficients *r*_-1_ by varying amplitude and phase of complex coefficient *A*. The amplitude of *r*_-1_ maintains 1 while the amplitude and phase of *A* change. In other words, the metagrating reflects all the incident light back when the light impinges from left side.


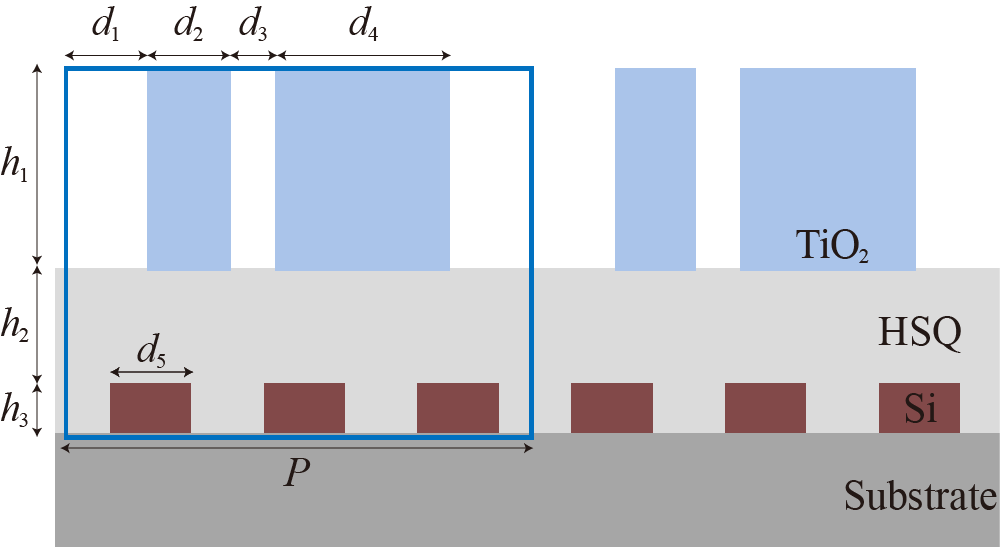


Fig. S4 The schematics and detailed parameters of the bilayer metasurface. The refractive indices of TiO_2_, HSQ, Si and substrate are 2.39, 1.42, 4.461-0.742×1*i* and 1.46, respectively. The system was illuminated by a TE-polarized plane wave. The parameters of metasurface are listed as follow: *d*_1_ = 110.5 nm, *d*_2_ = 91.5 nm, *d*_3_ = 46.8 nm, *d*_4_ = 190 nm, *d*_5_ = 83 nm, *h*_1_ = 416.9 nm, *h*_2_ = 397.5 nm, *h*_3_ = 183 nm, *P* = 532 nm.


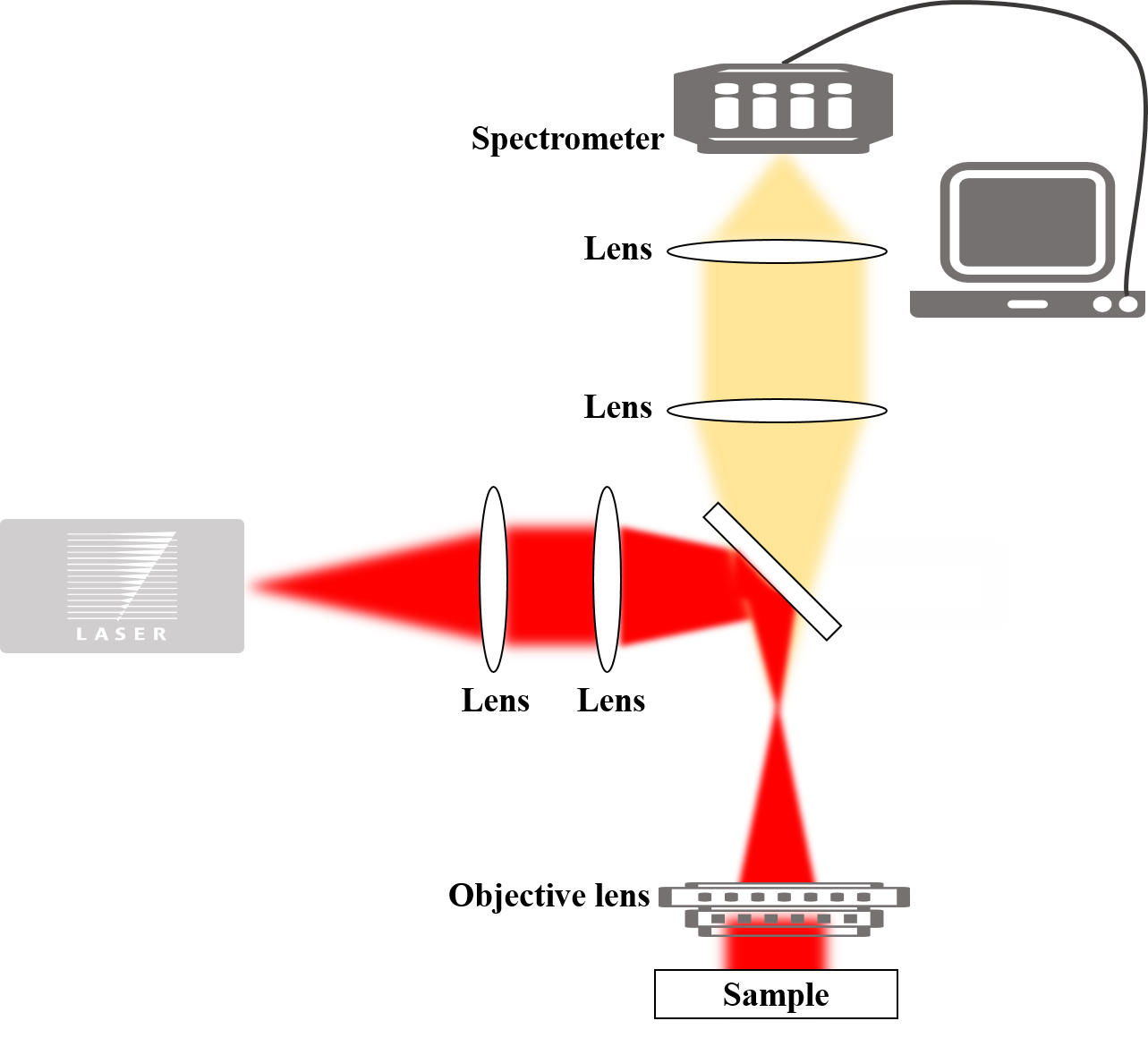


Fig. S5 The schematic of the angle-resolved spectrum system in micro-region. The measurement realizes angle resolution by focusing the reflective beam in the Fourier plane of the last lens. The incident angle is controlled by moving the position of the laser in the Fourier plane of the first lens. Then the intensities of +1^st^/−1^st^, and 0^th^ order light were detected for all wavelengths at once to obtain the reflection efficiency. The measurements were carried in a darkroom to improve the signal-to-noise ratio.

**References:**

1. Ito, K. & Iizuka H. Highly efficient -1st-order reflection in Littrow mounted dielectric double-groove grating. *AIP Advances* **3**, 062119 (2013).

2. Lalanne, P., Hugonin J. P. & Chavel P. Optical properties of deep lamellar Gratings: A coupled Bloch-mode insight. *J. Lightwave Technol.* **24**, 2442-2449 (2006).
